# Water depth affects reproductive allocation and reproductive allometry in the submerged macrophyte *Vallisneria natans*

**DOI:** 10.1038/s41598-017-16719-1

**Published:** 2017-12-04

**Authors:** Lei Li, Stephen P. Bonser, Zhichun Lan, Ligang Xu, Jiakuan Chen, Zhiping Song

**Affiliations:** 1 Center for Watershed Ecology, Institute of Life Science and Key Laboratory of Poyang Lake Environment and Resource Utilization, Ministry of Education, Nanchang University, Nanchang, 330031 China; 2 The Ministry of Education Key Laboratory for Biodiversity Sciences and Ecological Engineering, Institute of Biodiversity Science, Institute of Botany, Fudan University, Shanghai, 200438 China; 3 National Ecosystem Research Station of Jiangxi Poyang Lake Wetland, Nanchang, 330038 China; 4 Evolution and Ecology Research Centre, School of Biological, Earth and Environmental Sciences, University of New South Wales, Sydney, 2052 Australia; 5 Nanjing Institute of Geography and Limnology, Chinese Academy of Sciences, Nanjing, 210008 China

## Abstract

In freshwater ecosystems, shifts in hydrological regimes have profound effects on reproductive output (R), along with vegetative biomass (V) and survival of plants. Because reproductive allocation (RA) is allometric, it remains unclear whether the observed variation of RA in response to water level variability is due to fixed patterns of development or plasticity in the developmental trajectories. Here, we investigated shifts in RA of a submerged macrophyte *Vallisneria natans* in response to water depth to test the hypothesis that allometric trajectories of RA are highly plastic. Plants were grown at three water depths (50, 100 and 150 cm) and measured after 26 weeks of growth. The relationships between R and V among treatments were compared. Deep water affected both biomass and number of fruits produced per plant, leading to less sexual reproduction. Plants in deep water started flowering at a smaller size and despite their small mature size, had a relatively high RA. Furthermore, these plants had a much lower log R–log V relationship than shallow- or intermediate-water plants. In conclusion, reproduction of *V. natans* is highly variable across water depth treatments, and variations in reproductive allometry represent different strategies under an important stress gradient for these freshwater angiosperms.

## Introduction

Resource allocation to reproduction in plants is an important measure of their capacity to convert resources into propagules and thus a measure of fitness^1^. Patterns of reproductive allocation (RA) in plants are central to evolution of life histories and expression of ecological strategies^2^. Plants evolve patterns of RA in response to numerous selection pressures and constraints, including competition, disturbance and environmental stress^1,2^. For example, plants adapted to water-limited habitats tend to invest fewer resources in reproduction relative to vegetative growth, largely because flowering is water costly^3^. Therefore, variation in RA has a key role in plant adaptation to changing environments. Understanding the mechanisms underlying variation in RA is critical for predicting plant responses to future environmental changes. Here, we evaluate predictions on patterns and variation in RA of a submerged macrophyte in response to increased water depth from the perspectives of common development and plastic trajectory hypotheses.

Reproductive output is a function of RA – the proportion of biomass allocated into flowers and fruits relative to plant size^4^. Since plant growth is allometric, any factor that affects vegetative biomass (V) may also influence reproductive biomass (R). This size-dependent effect has been well documented^5–7^. In this event, patterns of RA can be better understood by analysing and interpreting allometric relationships between reproductive and vegetative investment within populations [i.e. reproductive (R) vs. vegetative (V) biomass, or log R vs. log V]. In several studies, there was large among population variation in the allometric slopes of the R–V relationship, with much of this variation induced by the biotic and abiotic environments in which individual plants were grown^8–11^. Contrary to this view, the relationship between R and V of a genotype is predicted to be not particularly plastic across different environments – rather, plasticity in this relationship is mostly due to differences in individual position along a common developmental trajectory^4,12^. However, this explanation has not been widely accepted. For example, it has been argued that some environmental stresses tend to favor flowering at relatively small sizes, and this plasticity can result in variability in the R–V relationship^6^. Analysing size-dependent reproduction is an important step in understanding plant biomass partitioning between vegetative and reproductive parts. However, more information is needed before we can predict patterns of RA in response to environmental changes.

Water depth is a key factor controlling the functional stability of aquatic systems^13,14^, largely because it has effects on growth, reproduction and re-establishment of submerged macrophyte species by changing irradiance to organs, and thus influencing carbon assimilation and nutrient use. These submerged species are important entities of freshwater ecosystems and constitute a dominant actor in many shallow lakes^15^. There is often zonation of submerged macrophytes along water-depth gradients. Generally, a certain level of water depth is conductive to maintenance of stable macrophyte communities^16^, whereas excessively shallow or deep water is detrimental to stability of submerged macrophyte communities^17,18^. Water depth in many aquatic systems is not always constant, but mainly depends on anthropogenic factors and regional conditions. Accordingly, investigating species-level responses to water depth is crucial for understanding the causes for community-level changes in changing environments.

Deeper water generally results in lower light intensity and higher water pressure but greater stability of other factors in the aquatic environment, e.g. temperature and sediment characteristics. Overall conditions for plant growth and sexual reproduction are less favourable in very deep water; consequently, reduced flowering and biomass of aquatic plants in deep-water habitats are common. For example, *Vallisneria* spp. use hydrophilous pollination and must produce female inflorescences that reach the water surface by elongation of its peduncles^19,20^. Therefore, reproductive investment may be limited to individuals that can invest the resources to make these large inflorescences. From an allocation perspective, the R–V allometric slope reflects how efficiently vegetative biomass is converted into reproductive biomass across plant sizes within a population^7,12^. Plant populations in more favourable environments may have a steeper slope for the log R–log V relationship, because under benign conditions, relatively large individuals can maximize RA with little risk to further growth or survival (i.e. high conversion efficiency at larger sizes). Conversely, for plants growing under certain forms of stress, high RA at small sizes may be favoured if mortality is so high that reducing early life reproduction and maintaining high allocation to growth can be hazardous (i.e. high conversion efficiency at smaller sizes). If so, populations adapted to deep-water habitats would have lower log R–log V slopes.

Hydrological regimes in Poyang Lake (the largest freshwater Lake in China) have been disturbed for decades by human activities, including dam constructions, sand mining, and drainage, with impacts on biodiversity and productivity^21–23^. Water depth is one of the most important factors influencing macrophyte community structure and distribution, at least at a local scale. The objective of this study was to identify effects of water depth in allocation strategies of *Vallisneria natans*, a dominant species in submerged macrophyte communities in the Poyang Lake. We grew *V. natans* at three water depths to address the following questions: 1) How do reproductive output and RAs of *V. natans* differ in response to water depths? 2) Is the slope of log R–log V relationships among individuals of this species greater in intermediate and shallow water treatments relative to the deep water treatment? 3) How does reproductive allometry among individuals of this species differ among various water depths?

## Results

### Effects of water depth on the growth of reproductive and non-reproductive ramets

**Figure 1:**
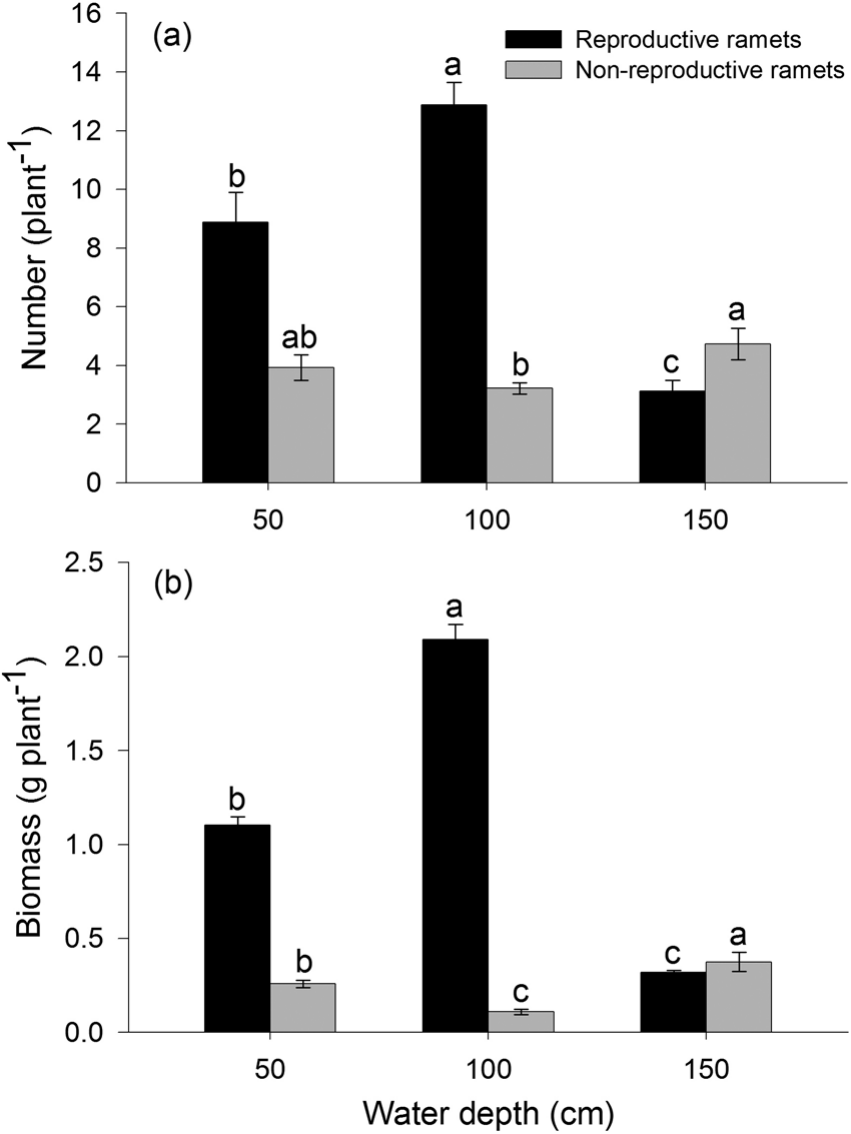
(**a**) Number and (**b**) biomass of reproductive and non-reproductive ramets of *Vallisneria natans* plants in the shallow, intermediate and deep water depth treatments (50, 100 and 150 cm, respectively). Values are means ± SE. Values with the same letter are not significantly different among water depth treatments at the *P* < 0.05 level.

Vegetative growth and reproduction had distinct responses to different water depths within our experimental populations of *V. natans*. Water depth had a strong effect on reproductive and non-reproductive ramet number, as well as total ramet number of plants (*P* < 0.001, *P* < 0.05 and *P* < 0.001, respectively). Plants grown at an intermediate depth (100 cm) had the most reproductive ramets, whereas those grown in deep water (150 cm) had the fewest (Fig. 1a). Furthermore, the fewest non-reproductive ramets occurred in intermediate water whereas deep water had the most (Fig. 1a). Plants in intermediate water had the greatest biomass of reproductive ramets and lowest biomass of non-reproductive ramets, with the opposite outcome for deep-water plants (Fig. 1b).

### Effects of water depth on plant biomass, height and reproductive traits

**Figure 2:**
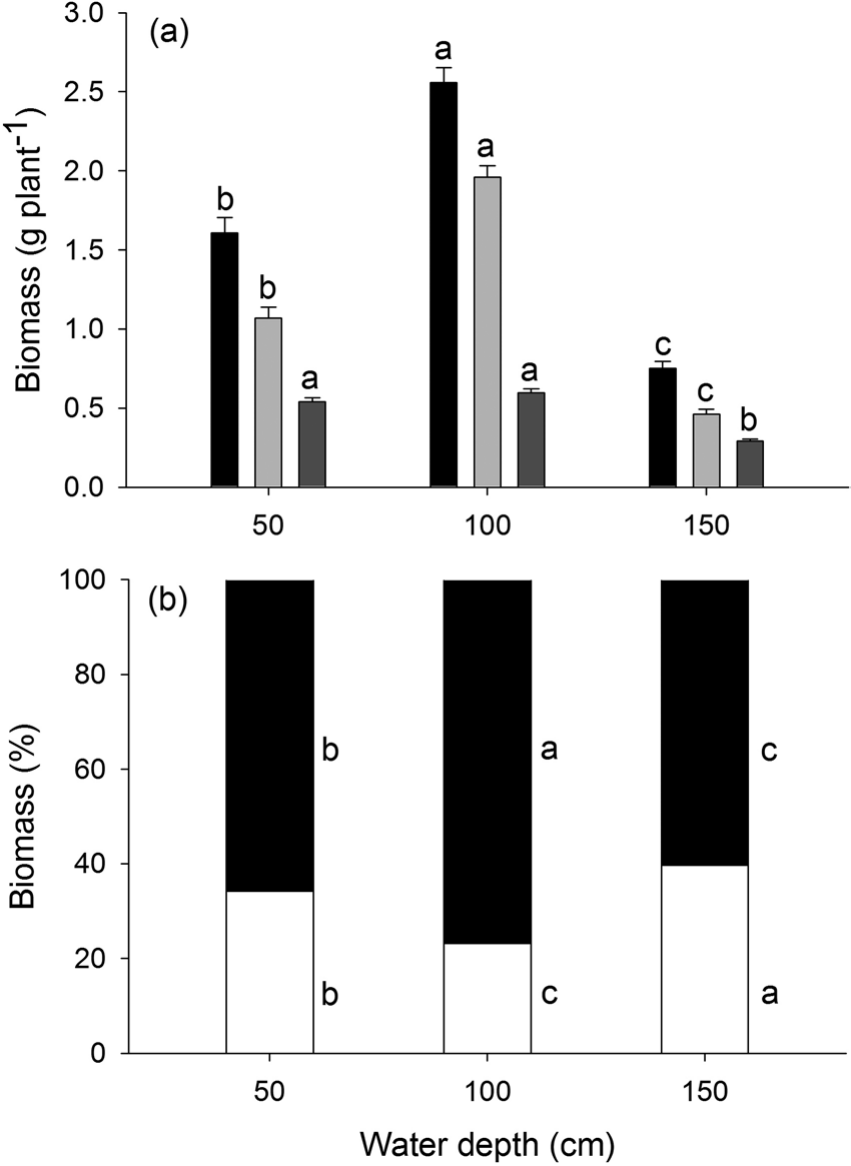
(**a**) Total biomass (black bars), vegetative biomass (light gray bars) and reproductive biomass (dark gray bars) and (**b**) biomass allocation to reproductive and vegetative (leaf + root + stolon) parts of *Vallisneria natans* plants (reproductive parts, white; vegetative parts, black) in the shallow, intermediate and deep water depth treatments (50, 100 and 150 cm, respectively). Values are means ± SE. Values with the same letter are not significantly different among water depth treatments at the *P* < 0.05 level.

Variations in water depth induced large individual variation in plant size and reproductive output. Vegetative mass and total biomass of *V. natans* were greatest in intermediate water, medium in shallow water and lowest in deep water (Fig. 2a). Plants in deep water had lower reproductive biomass than those in shallow or intermediate water (*P* < 0.001 for each), whereas there were no significant differences between shallow and intermediate water (*P* = 0.382; Fig. 2a). Log (vegetative mass), water depth effects and the water depth × log (vegetative mass) interaction were all highly significant in explaining variation in log reproductive mass (Table 1). Most of the variation in log (reproductive mass) produced by individuals was explained by variation in log (vegetative mass) (Table 1), indicating that plant size was the most important factor influencing reproductive output. The significant water depth × log (vegetative mass) interaction means that the populations in different water depth treatments expressed different relationships between reproductive and vegetative mass (see below).

**Table 1:** General linear model of the effects of water depth and log (vegetative mass) on log (reproductive mass). Adjusted *r* ^2^ for the model is 0.96.

Source	SS	df	*F*	*P*
Water depth	0.115	2	50.4	<0.001
Log (vegetative mass)	0.317	1	278.1	<0.001
Water depth × log (vegetative mass)	0.044	2	19.2	<0.001

**Figure 3:**
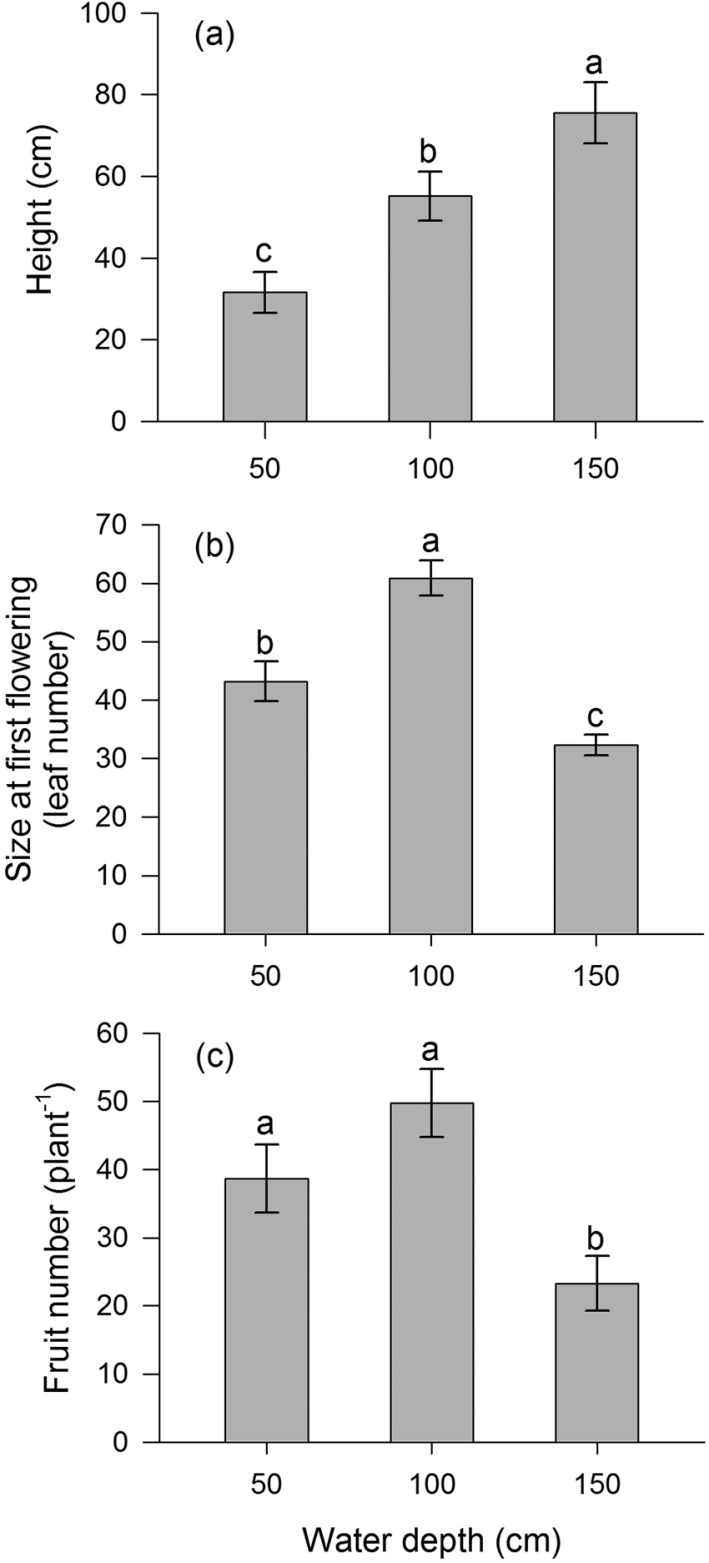
Effects of water depth on (**a**) height, (**b**) leaf number at the initiation of reproduction and (**c**) fruit number of *Vallisneria natans*. Values are means ± SE. Values with the same letter are not significantly different among water depth treatments at the *P* < 0.05 level.

Mean plant height increased sharply with increasing water depth (*P* < 0.001; Fig. 3a). In deep water, size at first flowering (measured as leaf number at the initiation of reproduction) and the number of fruits were smaller than for plants grown in shallow or intermediate water (*P* < 0.01; *P* < 0.001; Fig. 3b,c). Plants in shallow water also initiated flowering at smaller sizes than those in intermediate water (Fig. 3b). Surprisingly, shallow-water plants also produced fewer fruits than intermediate-water plants, though this was not significant (Fig. 3c).

### Biomass allocation to vegetative growth and reproduction

In a traditional analysis of the RA at the population level as a function of water depth, percent allocation to reproductive biomass and vegetative biomass of the species varied significantly among treatments (*P* < 0.001; *P* < 0.001; Fig. 2b). Plants in deep water had a relatively high allocation to reproduction compared to those in the two other water treatments, whereas plants in shallow water had higher allocation to reproduction than those in intermediate water (Fig. 2b). The biomass invested to vegetative parts (leaf + root + stolon) was lowest for plants in deep water, medium for plants in shallow water and highest for plants in intermediate water (Fig. 2b).

### Reproductive allometric relationships among individuals

**Figure 4:**
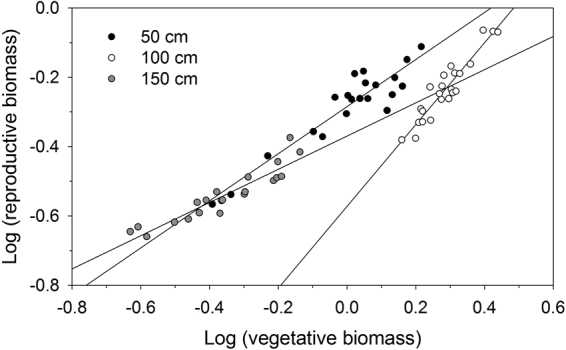
The relationships between log (reproductive biomass) and log (vegetative biomass) among individuals of *Vallisneria natans* grown in the shallow, intermediate and deep water depth treatments (50, 100 and 150 cm, respectively), with standardized major axis regression lines for each treatment.

There were significant positive relationships between reproductive biomass and vegetative biomass at all water depths (Table 2; Fig. 4). Water depth significantly affected SMA slopes of log R–log V relationships (*P* < 0.01). The *post hoc* multiple comparisons showed that the log R–log V slope was highest in intermediate water, where the slope was significantly greater than 1 (the slope of isometry) and significantly greater than the other treatments (Table 2; Fig. 4). Shallow-water plants also had a higher log R–log V slope than deep-water plants (*P* < 0.01; Table 2; Fig. 4), although SMA slopes in shallow- and deep-water treatments were significantly less than 1 (Table 2).

**Table 2:** Estimated parameters in allometric regression between log (reproductive biomass) and log (vegetative biomass) of *Vallisneria natans* grown at three levels of water depth, using Standardized Major Axis.

Water depth (cm)	Slope	95% CI	Intercept	R^2^
50^b^	0.73**	0.61–0.86	−0.29	0.87
100^a^	1.25**	1.08–1.46	−0.59	0.89
150^c^	0.51***	0.43–0.61	−0.36	0.87

Asterisks represent slopes that are significantly different from 1: **P < 0.01, ***P < 0.001. Treatments within a column with different letters are significantly different at the *P* < 0.05 level.

## Discussion

Allocation to reproduction in *V. natans* was highly variable across water depth treatments, and variation in RA represents distinct strategies under an important stress gradient for these plants. Most importantly, the experimental design enabled us to determine causes of variation in RA by using a system that should highly favour plasticity in developmental trajectories that would not be consistent with variability along a common developmental trajectory.

Reproductive ramet number was a major factor affecting reproductive output in clonal macrophyte *V. natans*. For many annual plants, architectural traits are likely to be important for seed production^24^, as plants must first accumulate resources and build reproductive machinery (ramets or branches) before any products (seeds) can be produced^12^. Trade-offs are very difficult to actually demonstrate in clonal plants^25^. In the present study, plants with a high number of reproductive ramets tended to have few non-reproductive ramets. This suggests a trade-off between reproductive and vegetative ramets across environments. In addition, this could suggests a change as plants develop – where they can, they convert ramets to reproduction. So plants become more reproductive and less vegetative, giving the appearance of a trade-off.

Light availability along the water depth gradient exerts a primary influence on the growth of submerged macrophytes, mainly in terms of photosynthesis. Light attenuates very quickly in water, and the decrease of light availability in water is considered the main reason for the extinction of submerged plants^26^. Due to vertical light attenuation in water, plants in deep water tended to reduce their resource investment in ramet production, and prioritize vertical growth and elongation of leaves, which would confer greater uptake of light. In contrast, with plant height of 31.5 cm at a water depth of 50 cm, it was very likely that leaves on plants in the shallow water treatment had access to very high photosynthetically active radiation. In that case, in shallow water, high light conditions might be a stressor for submerged macrophytes due to photo-inhibition^27^. Therefore, plants in shallow water seemed to priorize lateral growth in lieu of height growth. Furthermore, in the present study, plants invested much fewer resources to reproductive ramets in deep versus shallow or intermediate water. We inferred that light limitation inhibited the number of meristems that could potentially produce reproductive structures for *V. natans*, negatively influencing the number of inflorescences and fruits produced. An important reason for this is that production of a single successful inflorescence in deep water is much more expensive in terms of biomass allocation due to its length, as an inflorescence that does not reach the surface of the water will not receive pollen.

Water depth had major effects on plant size and reproductive output. In a previous study, *V. natans* attained maximum total biomass and leaf mass at 60 cm, whether in clay or sandy loam^28^. However, in the present study, maximum biomass of *V. natans* was obtained at 100 cm, perhaps due to water clarity. Generally, macrophytes only grow in restricted ranges of water depth, resulting in a zonal distribution. Reproductive output (mass of fruits produced) of *V. natans* was reduced by deep water, since production of very long peduncles in deep water limits fruit or seed production (i.e. if plants can only allocate so much to reproduction, and most of it is in the peduncle, then this constraint on RA may limit fecundity). It is remarkable that shallow-water plants also produced fewer fruits than intermediate-water plants ‒ though this was not significant. Perhaps there are potentially high costs associated with being tall in shallow water, where light intensity is extremely strong. If stressful conditions for plant growth and survival at the apical meristems intensify as plants get larger, the ability to produce, maintain and mature reproductive structures would also decrease as plants get larger. Lower sexual reproduction in the submerged angiosperm may influence seed dispersal, re-establishment after disappearance, and may also reduce resistance to other environmental stressors due to lower genetic diversity^29^.

Greater reproduction allocation is typically associated with exposure to stress. Many factors can severely limit reproduction but selection can still favour a high commitment to reproduction^2^. In this study, individuals of *V. natans* growing in deep water (the high stress treatment) invested more resources to reproductive structures and initiated reproduction at smaller sizes than those growing in shallower water. According to the life history theory, individuals that start reproducing earlier in life or increase resource allocation to reproduction are usually favoured under harsh environments, due to reduced life expectancy^30^. We inferred that low light level in deep water can trigger flowering or result in higher RA, as plants have been selected to reproduce as much as it can with the resources available when the probability of surviving or growing large is low. In this case, delaying reproduction and maintaining high allocation to vegetative growth would be inherently risky, as plants may not reach an optimal size for reproduction before the end of a limited growing season. High RA under adverse conditions could be of great benefit to *V. natans*, which often faces large variations in water depth over time and space in Poyang Lake.

In shallow water, where reproduction has a lower cost due to production of smaller inflorescences (i.e. a lower investment in peduncles to reach the water surface), plants had higher allocation to reproduction than intermediate-water plants. This difference in reproduction allocation may be also an adaptive response to limits imposed on plant growth and survival by low water level. Plants in low water also may at risk of drying. Small reductions in water level during a summer dry season could cause mortality in shallow-water plants, but not in intermediate-water plants. Additionally, if selection is on fecundity (seed production), plants in shallow water may produce more seeds per unit reproductive mass since they produce short peduncles. The ability to reproduce under such potentially stressful conditions is crucial for this annual freshwater angiosperm, and fecundity selection could maximize fitness in conditions where non-seed reproductive allocation is low.

Allometry can be used to assess effects of size on expression of allocation to a given trait or plant function. The log R–log V relationship was highly plastic (significantly different allometric slopes across treatments), indicating that a size-dependent effect had an important role in variations of resource investment in reproduction of *V. natans* in response to variable water depths. A size-dependent RA trajectory has been considered as a bet-hedging strategy that ensures that the plant species converts certain plant growth to reproduction under changing environments to improve the species’ fitness^12^. Differences in size-dependent RA may be an adaptive response to environmental limitation on plant growth and survival in unfavourable environments. *V. natans* plants exhibited a more positive slope for the log R–log V relationship under relatively favourable conditions (100 cm depth), because relatively large plants can maximize RA with little risk to further growth or survival whereas small individuals developed more slowly and had fewer reproductive ramets and lower RA at harvest than large individuals. Rather, for this annual plant growing in deep water, lower log R–log V slopes are favoured as high RA at small size may be advantageous if mortality due to limited light is so high that delaying reproduction and maintaining high allocation to growth may be hazardous. However, for plants growing in shallow water, if the environment prevents large individuals from producing proportionally more offspring than small individuals because additional tissues are damaged by the stressful environment (excessive light), the log R–log V slope would be lower. In this sense, a change in allometric slope represents a trade-off of performances at larger versus at smaller sizes.

An alternative explanation for plasticity in trajectories of RA is that the relationship between vegetative and reproductive biomass seen across plants at final development was a product of plasticity in size at first reproduction^6^. *V. natans* responded to stressful environments (deep or shallow water) by decreasing their reproductive threshold, that is, by an earlier flowering and fruiting. This response suggests that even the major developmental stages such as the size at reproduction can be highly plastic. Experiments on herbaceous and woody plants demonstrated that stressful environments - namely resource impoverishment and competition – induce reproduction at smaller sizes^2,31,32^. Our results were consistent with these patterns. The adaptive value of reproduction at larger sizes in benign environments relies on a positive relationship between fecundity and size at reproduction, so that attaining a larger size implies an increased life time reproductive output^33^.

In conclusion, deep water resulted in lower sexual reproduction but higher RA in this freshwater angiosperm species; this has implications for population persistence, species dispersal and evolution. Based on a comparative allometric approach, plants growing along a water-depth gradient had distinct patterns of size-dependent resources allocation, suggesting that reproductive allometry can be an adaptive strategy of plant growth and allocation, rather than a product of fixed developmental constraints. Plasticity in reproductive strategies in macrophytes is likely to be of adaptive significance under future shifts of hydrological regimes due to human disturbance and regional climate change.

## Methods

### Plant species

We examined reproductive allometry in *Vallisneria natans* (Lour.) Hara (Hydrocharitaceae). *Vallisneria* is a monocot genus of submerged dioecious species that grows at the bottom of freshwater bodies and undergoes hydrophilous pollination on the water surface^20^. *V. natans* is a stoloniferous submerged macrophyte, which usually forms monodominant communities in many freshwater ecosystems from northeast to southwest China. It is frequently, though not always, an annual species^34^. The species have rosette-like ramets with multiple linear leaves 20–200 cm long and 0.5–2 cm wide. New ramets are produced through extension of stolons. Female flowers are connected to the mother plant by a long spiral peduncle that forms in a leaf axil, where as male inflorescences, containing hundreds of minute (0.5 mm) flowers enclosed by a short-peduncled spathe, release their flower as small “boats” which rise to the water surface. Flowering and seed set are indeterminate, occurring from July to October in eastern China. This species also has an important role in the structure and function of freshwater ecosystems, such as purifying water and providing food and habitat for aquatic fauna, and is therefore used frequently to restore shallow lakes. It was reported that *V. natans* had high phenotypic variation and great potential to adapt to highly variable environments^34^.

### Experimental design

Mature fruits of *V. natans* were collected in late October, 2014 from female plants growing naturally at the water depths of 80–100 cm in Meixi Lake (116°03′E, 29°13′N), a shallow lake within Poyang Lake National Nature Reserve (115°55′–116°03′E, 29°05′–29°15′N), Jiangxi Province, China. The water table in Meixi Lake may fluctuate, resulting in the plants being at a depth range of 20–180 cm. Upon collection, the flesh and pectin of each fruit were carefully removed, and seeds were stored in a plastic container filled with water, in darkness at 4 °C for 5 months.

The experiment was conducted in a mesocosm facility located at the Poyang Lake Laboratory for Wetland Ecosystem Research, Chinese Academy of Sciences (116°03′E, 29°26′N), Lushan City, in the northwestern part of Poyang Lake Basin of China. In April 2015, over 300 seeds of *V. natans* were germinated in each of three bins (56 cm long × 38 cm wide width × 28.5 cm deep) containing sterilized Poyang Lake sediment (~10 cm thick) and 10 cm of water. In late May 2015, when seedlings had produced four or five leaves, we randomly transplanted one seedling into each of 225 pots (18 cm diameter × 12 cm deep). Each pot contained 10 cm Poyang Lake sediment (TN: 2.41 mg g^−1^, TP: 0.75 mg g^−1^, organic matter content: 5.82%). After transplantation, the experiment was conducted in 9 outdoor mesocosms (2.0 m long × 2.0 m wide × 1.6 m deep) arranged in a randomized block design with three repetitions per water depth treatment. That is, one of three water depth treatments was randomly assigned in each of the three replicate blocks. According to the field investigation over 25 lakes along the middle-lower reaches of Yangtze River, the range of water depth at which *V. natans* naturally distributed was approximately 0.2–1.5 m^35^, although this species may occurred at much deeper water in other water bodies such as Lake Erhai and Fuxian where the water is much clearer than that of those lakes in the middle-lower reaches of Yangtze River. According to this, three levels of water depth were used: 50 cm (shallow), 100 cm (intermediate) and 150 cm (deep). The mesocosms were fully filled with fresh lake water (TN: 1.49 mg l^−1^; TP: 0.06 mg l^−1^). The water was filtered using a plankton net (pore size: 0.505 mm) to exclude aquatic animals. Twenty-five pots of planted *V. natans* were placed in each mesocosm at 50 cm depth for 10 d acclimation, then were suspended at the appropriate water depth by nylon ropes connected to platforms that constructed from tubes of galvanized metal lying on top of the mesocosms. Water was supplied to each mesocosm from an irrigation reservoir adjacent to the mesocosm facility. Additional water was added to the mesocosms two or three times per week to keep the water level stable and aid in water circulation. Throughout the experiment, observed phytoplankton on the water surface was removed using a filter net (pore size: 0.03 mm), and epiphyton on plants was removed using a soft brush.

### Harvest and data collection

Treatments were maintained over 18 weeks from June to October 2015. We did not focus on male individuals in this study due to the problem of quantifying reproductive biomass for males that released their minute flowers to the water surface at maturation. During this experiment, plant height, leaf number and fruit numbers were recorded biweekly. Height was measured as the distance from the soil surface to the apex of the longest leaf (to the nearest 1 mm). Size at first flowering was recorded as the number of leaves at the initiation of reproduction^32^. Besides some plants died during the experiment (n = 31), the total number of the pots having plants was 194. Among these, we did not include the plants of which leaves were grazed by unidentified aquatic insects in trait measurement (n = 34). Therefore, the final numbers of female plants were 21, 23 and 21 for the three water depths, respectively. On 5 October 2015, when all *V. natans* individuals were fully mature and started to senesce (end of flowering), they were harvested. Pots with healthy individuals with intact leaves were removed from the tanks and each plant was hand-washed. Each plant was separated into two types of ramets by aboveground part: reproductive ramets and non-reproductive ramets (already mature but no flower), and their numbers were recorded. Reproductive ramets were further divided into leaves, peduncles and fruits. Therefore, dry mass of the two types of ramets were determined separately. Also, each plant has two parts of biomass: vegetative part and reproductive part. Vegetative biomass (V) included leaves, stolons and roots from both reproductive ramets and non-reproductive ramets, and reproductive biomass (R) included peduncles and fruits. All separated components of each plant were put into individual paper bags, oven-dried at 70 °C for 72 h and then weighed.

### Statistical analyses

General linear mixed model was used to analyse variations of all plant traits (height, total biomass, vegetative biomass, fruit mass, number of leaves at first flowering, fruits, reproductive ramets and non-reproductive ramets) and allocations to different components in response to water depths. Comparisons between means were done with a LSD test, with significance level of *P* < 0.05. Effects of water depth and log V on log R were tested with general linear models, with log R as the response variable and water depth and log V as variables. A univariate analysis was used to test for interactions between water depth treatment and log V. All data were analysed with SPSS statistical software (version 19.0; SPSS Inc., Chicago, IL, USA).

To analyze allometric scaling in biomass patterns and to homogenize variance, biomass values were log-transformed. Allometric relationships between R and V among individuals were analysed by the log transformed version of the classical “allometric” model: log R = *a* log V + log *b*, where *a* is the allometric slope and *b* is the allometric coefficient. Standardized major axis (SMA) regression was used to determine the R–V relationship at harvest under different water depth treatments, using the SMATR package in R version 3.11^36^. We tested whether the slope among individuals of each treatment was different from 1. We tested whether there were significant differences in slopes among water depth treatments by running multiple post hoc comparisons. The significance level for testing slope heterogeneity and difference from slope = 1 was *P* < 0.05.
